# Economic burden of colorectal and breast cancers attributable to lack of physical activity in Brazil

**DOI:** 10.1186/s12889-021-11221-w

**Published:** 2021-06-22

**Authors:** Leandro F. M. Rezende, Gerson Ferrari, Luciana Ribeiro Bahia, Roger Dos Santos Rosa, Michelle Quarti Machado da Rosa, Romulo Cristovão de Souza, Dong Hoon Lee, Edward Giovannucci, José Eluf-Neto

**Affiliations:** 1 Universidade Federal de São Paulo, Escola Paulista de Medicina, Departamento de Medicina Preventiva, Rua Botucatu, 740 -Vila Clementino, Sao Paulo, SP 04023-062 Brazil; 2 Universidad de Santiago de Chile (USACH), Escuela de Ciencias de la Actividad Física, el Deporte y la Salud, Santiago, Chile; 3 National Institute of Science and Technology for Health Technology Assessment (IATS), Universidade do Estado do Rio de Janeiro (UERJ), Rio de Janeiro, Brazil; 4 National Institute of Science and Technology for Health Technology Assessment (IATS), Departamento de Medicina Social, Universidade Federal do Rio Grande do Sul, Porto Alegre, Brazil; 5 Departamento de Tecnologias da Informação e Educação em Saúde, Faculdade de Ciências Médicas – UERJ, Rio de Janeiro, Brazil; 6 Department of Nutrition, Harvard T.H. Chan School of Public Health, Boston, MA USA; 7 Department of Epidemiology, Harvard T.H. Chan School of Public Health, Boston, MA USA; 8 Channing Division of Network Medicine, Department of Medicine, Brigham and Women’s Hospital and Harvard Medical School, Boston, MA USA; 9 Departamento de Medicina Preventiva, Faculdade de Medicina FMUSP, Universidade de Sao Paulo, Sao Paulo, SP Brazil

**Keywords:** Physical inactivity, Cancer, Brazil, Low- and middle-income countries

## Abstract

**Background:**

The increasing number of cancer patients has an escalating economic impact to public health systems (approximately, International dollars- Int$ 60 billion annually in Brazil). Physical activity is widely recognized as one important modifiable risk factor for cancer. Herein, we estimated the economic costs of colon and post-menopausal breast cancers in the Brazilian Unified Health System (SUS) attributable to lack of physical activity.

**Methods:**

Population attributable fractions were calculated using prevalence data from 57,962 adults who answered a physical activity questionnaire in the Brazilian National Health Survey, and relative risks of colon and breast cancer from a meta-analysis. Annual costs (1 Int$ = 2.1 reais) with hospitalization, chemotherapy and radiotherapy were obtained from the Hospital and Ambulatory Information Systems of the Brazilian SUS. Two counterfactual scenarios were considered: theoretical minimum risk exposure level (≥8000 MET-min/week) and physical activity guidelines (≥600 MET-min/week).

**Results:**

Annually, the Brazilian SUS expended Int$ 4.5 billion in direct costs related to cancer treatment, of which Int$ 553 million due to colon and breast cancers. Direct costs related to colon and breast cancers attributable to lack of physical activity were Int$ 23.4 million and Int$ 26.9 million, respectively. Achieving at least the physical activity guidelines would save Int$ 10.3 mi (colon, Int$ 6.4 mi; breast, Int$ 3.9 mi).

**Conclusions:**

Lack of physical activity accounts for Int$ 50.3 million annually in direct costs related to colon and post-menopausal breast cancers. Population-wide interventions aiming to promote physical activity are needed to reduce the economic burden of cancer in Brazil.

## Background

Cancer is the second leading cause of death and disability-adjusted life years in Brazil [1]. Breast and colorectal cancer are among the most common cancers, with an estimated combined number of 137,413 new cases and 42,924 deaths in 2018 [2]. By 2040, breast and colorectal cancers are expected to increase over 50% due to demographic and lifestyle changes [2]. Such an increase in the cancer burden may put further pressure on an already overwhelmed Brazilian Unified Health System (*Sistema Único de Saúde* - SUS), which covers more than 75% of the population [3, 4].

Physical activity is associated with lower risk of several types of cancer [5]. In 2018, the World Cancer Research Fund (WCRF) concluded that strong evidence supports that physical activity reduces the risk of colon, breast and endometrial cancers [6]. Nonetheless, dose-response relationship has been well-characterized for colon and postmenopausal breast cancers only [7]. Of note, global estimates suggest that lack of physical activity causes 10% of all breast and colons cancers [8]. To reduce the risk of these cancers, as well as other non-communicable diseases, the 2020 World Health Organization (WHO) guidelines for physical activity and sedentary behaviour calls for adults to do 150–300 min of moderate intensity, or 75–150 min of vigorous-intensity aerobic physical activity per week, or any equivalent combination of the itensities [9]. Despite the well-documented health benefits of reaching physical activity guidelines, global physical activity levels are not sufficient. In 2016, age-standardized prevalence of insufficient physical activity was 27.5%, with a higher prevalence in women (31.7%) than in men (23.4%) [10]. Latin America and Caribbean countries have showed the highest prevalence of insufficient physical activity (39.1%) worldwide, and Brazil presented the highest prevalence (47%) within the continent [10].

Insufficient physical activity cost health-care systems international dollars (Int$) 53.8 billion worldwide in 2013 [11]. Of note, these estimates were calculated considering direct health-care costs, productivity losses, and disability-adjusted life-years for coronary heart disease, stroke, type 2 diabetes, breast cancer, and colon cancer attributable to insufficient physical activity [11]. In Brazil, it has been estimated that approximately 10 thousand cancer cases and 3 thousand cancer deaths per year are attributable to insufficient physical activity [12]. However, to our knowledge, the economic costs of cancer in Brazil attributable to lack of physical activity are unknown, besides its potential to inform the financial impact of this exposure on the health system [13]. A cost-of-illness study include direct costs to health systems, patients and their families, and more broadly, the indirect costs to society (absenteeism, premature retirement and death). Nonetheless, data on indirect costs of cancer to society are unavailable in Brazil. On the other hand, a recent study estimated that approximately Int$ 7.54 billion were spent with oncological treatment by the Brazilian federal government from 2001 to 2015, of which Int$ 3.24 billion with breast cancer patients and Int$ 1.39 billion with colorrectal cancer patients [14].

In this study, we estimated the direct health care costs of colorectal and breast cancers in the Brazilian SUS attributable to lack of physical activity.

## Methods

We designed a cost-of-illness study to estimate the direct costs of colorectal and breast cancers attributable to lack of physical activity from the perspective of the Brazilian SUS. This approach uses aggregated disease costs along with potential impact fraction (PIF) estimates to calculate the costs attributable to a given risk factor [13].

### Direct health care costs of colorectal and breast cancers

Direct health care costs of all cancer (C00-C97), colorectal cancer (C18-C20), colon (C18), breast cancer (C50), and postmenopausal breast cancer (C50 for women 50 years or older) were obtained from the Brazilian SUS Ambulatory (Outpatient) Information System (SIA/SUS) [15] and the Hospital (Inpatient) Information System (SIH/SUS) [16] in 2017 (herein considered the average of 2015–2017) based on the International Classification of Diseases, 10th Revision (ICD-10). SIH/SUS and SIA/SUS are publicly available and contain deidentified inpatient and outpatient care data. Direct health care costs were defined as those of outpatient and inpatient procedures. In our study, we included the following procedures and costs for patients aged 20 years or older:

Outpatient costs: chemotherapy (eg., conventional chemotherapy, targeted therapy, hormone therapy, immunotherapy and supportive therapy) and radiotherapy.

Inpatient costs: surgery and other hospital costs (eg., diagnostic and clinical procedures and organ, tissue and cell transplantation, including chemotherapy during hospitalization).

We converted the monetary values in Reais (R$) to Int$, considering the purchasing power parity (PPP) for 2015–2017 (conversion factor 2.10) [17].

### Physical activity assessment

Physical activity was obtained from a national representative health survey conducted in Brazil in 2013, the National Health Survey (*Pesquisa Nacional de Saúde* – PNS 2013). Details about PNS methods have been reported elsewhere [12]. In this study, we included 57,962 adults aged 20 years or older that responded to a questionnaire about frequency and duration of recreational, occupational, commuting to work, commuting to other daily activities, and household activities in a typical week. We assigned metabolic equivalent of tasks (MET) for each activity and summed them to obtain total volume of physical activity (MET-min/week). MET were used to weight different types of aforementioned activities according to its intensity (meaning higher intensity having higher weight), as per the 2011 compendium of physical activities [18]. Details for these methods has been described elsewhere [12].

All PNS data are available on the Brazilian Institute of Geography and Statistics (*Instituto Brasileiro de Geografia e Estatística*, IBGE) website at: http://www.ibge.gov.br/home/estatistica/populacao/pns/2013/default_microdados.shtm. The PNS was approved by Brazil’s National Research Ethics Committee (*Comissão Nacional de Ética em Pesquisa*, CONEP) with the National Health Council (*Conselho Nacional de Saúde*) Resolution No. 466/12 (No. 328159, June 26th, 2013), and all participants signed an informed consent at interview.

### Data analysis: cost-of-illness modelling

To estimate the direct health care costs of breast and colorectal cancers in the Brazilian SUS attributable to lack of physical activity, we first estimated PIF. Physical activity has been consistently associated with colon (C18) and postmenopausal breast cancer (C50 for women 50 years or older) [7]. Therefore, we first calculated PIF for colon by sex, and PIF for postmenopausal breast cancer for women using physical activity data from PNS 2013 and relative risks (RR) from published meta-analysis [7]:

$$ PIF=\frac{\sum_{i=1}^n{P}_i{RR}_i-{\sum}_{i=1}^nP{\hbox{'}}_i{RR}_i}{\sum_{i=1}^n{P}_i{RR}_i} $$

Pi = proportion of the population at the level *i* of physical activity categories;

*P’*_*i*_ = proportion of the population at the level *i* of physical activity categories in the counterfactual scenario. In this we considered two counterfactual scenarios: (1) Theoretical minimum risk exposure level (TMRE): population reaching ≥8000 MET-min/week– aka population attributable fraction (PAF); (2) Physical activity guidelines (PA guidelines): population reaching at least 600 MET-min/week.

*RR*_*i*_ is the relative risk of postmenopausal breast cancer and colon cancer at the level *i* of physical activity categories. These RR values are currently used in the Global Burden of Disease study [7].

Levels *i* of physical activity were < 600, 600 to 3999, 4000 to 7999, and ≥ 8000 MET-min/week (reference group), same in the aforementioned dose-response meta-analysis [7].

PIFs were applied to procedures and costs of hospitalizations, chemotherapy and radiotherapy of colon cancer and postmenopausal breast cancer to calculate the costs attributable to lack of physical activity. Then, we divided colon cancer (C18) and postmenopausal breast cancer (C50 for women 50 years or older) costs attributable to lack of physical activity by total colorectal (C18-C20) and breast cancer (C50) costs, respectively. Data analysis were performed in Stata 15.0 and Microsoft Excel Office® 2007 spreadsheets.

## Results

Table 1 displays the costs of hospitalization, chemotherapy and radiotherapy by cancer site and sex in Brazil in 2017. Approximately Int$ 4.5 billion was spent on direct health care related to all cancer types, of which 12.4% or Int$ 553 million were due to colorectal cancer (Int$ 212 million) and breast cancer (Int$ 341 million). For colorectal cancer, Int$ 121 million were spent on chemotherapy, Int$ 82 million on hospitalization, and Int$ 8 million on radiotherapy. Colorectal cancer costs were similar for men (Int$ 105 million) and women (Int$ 106 million). For breast cancer, Int$ 231 million were spent on chemotherapy, Int$ 62 million on hospitalization, and Int$ 48 million on radiotherapy.

**Table 1:** Direct public health care procedures and costs for breast cancer, colorectal cancer, and all cancer in Brazil, 2015–2017^a^

Procedures, sex	Breast cancer			Colorectal			All cancers		
	Number of Procedures	Costs (R$)	PPP	Number of Procedures	Costs (R$)	PPP	Number of Procedures	Costs (R$)	PPP
**Hospitalization**									
Both	61,990	131,229,010	62,450,354	67,168	173,310,696	82,476,537	1,598,494	3,820,831,624	1,818,289,161
Men	NA	NA	NA	34,128	86,886,272	41,348,162	766,068	1,833,678,694	872,626,282
Women	61,990	131,229,010	62,450,354	33,040	86,424,424	41,128,374	832,426	1,987,152,930	945,662,879
**Chemotherapy**									
Both	1,509,410	485,706,784	231,142,188	154,875	254,701,471	121,209,457	8,917,781	4,321,452,359	2,056,528,724
Men	NA	NA	NA	79,376	125,324,520	59,640,476	3,230,152	1,781,980,080	848,023,515
Women	1,509,410	485,706,784	231,142,188	75,500	129,376,951	61,568,980	5,687,629	2,539,472,279	1,208,505,209
**Radiotherapy**									
Both	66,998	100,279,940	47,722,053	12,414	17,258,377	8,213,060	821,893	1,227,072,660	583,949,552
Men	NA	NA	NA	6746	9,444,164	4,494,367	373,662	565,389,306	269,062,170
Women	66,998	100,279,940	47,722,053	5668	7,814,214	3,718,693	448,231	661,683,353	314,887,383
**Total**	1,638,398	717,215,734	341,314,594	234,457	445,270,545	211,899,054	11,338,168	9,369,356,643	4,458,767,438

*PPP* Purchasing power parity in 2015–2017 (conversion factor 2.10). ^a^Average of costs in 2015–2017

**Fig. 1:**
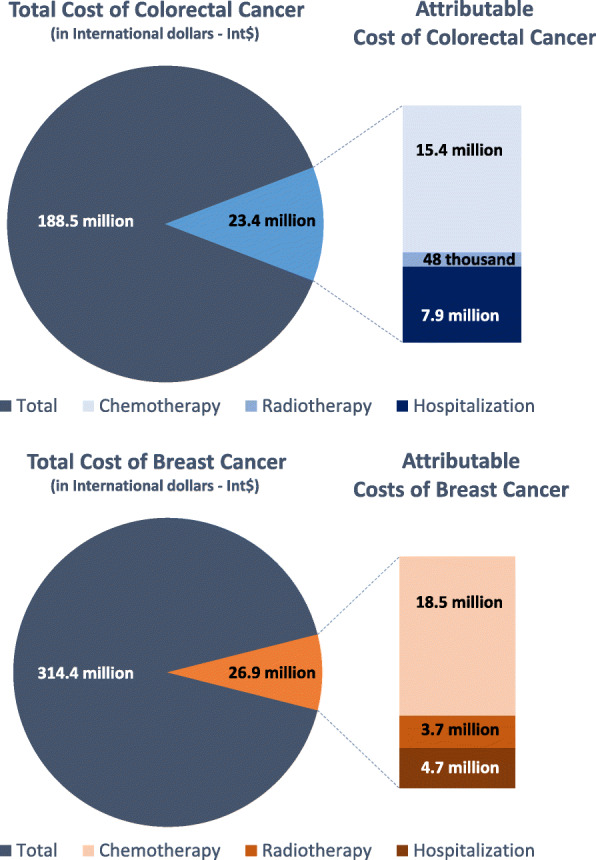
Total and attributable direct public health care costs of breast and colorectal cancers in the Brazilian Health System attributable to lack of physical activity, 2015-2017*

Direct costs with colon cancer (Int$ 134 million) represented 63% of all colorectal cancer costs (Fig. 1 and Table 2). Considering the TMRE scenario, about Int$ 23 million of colon cancer costs were attributable to lack of physical activity, which represented 11% of all colorectal cancer costs. Attributable costs with colon cancer (Table 2) were slightly higher in women (Int$ 12 million) than in men (Int$ 10 million). Most of the attributable costs were due to chemotherapy (Int$ 15 million), followed by hospitalization (Int$ 8 million) and radiotherapy (Int$ 48 thousand). In the PA guidelines scenario, we estimated that Int$ 6 million could be potentially saved annually by increasing population-wide physical activity level to ≥600 MET-min/week, of which Int$ 4 million on chemotherapy, Int$ 2 million on hospitalizations and Int$ 13 thousand on radiotherapy.

**Table 2:** Attributable Direct public health care procedures and costs for colon cancer by increasing physical activity in Brazil, 2015–2017^a^

	–			TMRE (≥8000 MET-min/week)			PA guidelines (≥600 MET-min/week)		
Procedures, sex	Total			Attributable			Attributable		
	Number of Procedures	Costs (R$)	PPP	Number of Procedures	Costs (R$)	PPP	Number of Procedures	Costs (R$)	PPP
**Hospitalization**									
Both	43,061	95,493,424	45,444,206	7506	16,648,732	7,922,937	2045	4,522,081	2,152,006
Men	21,320	46,235,648	22,003,005	3549	7,696,932	3,662,880	960	2,080,133	989,911
Women	21,741	49,257,772	23,441,199	3957	8,951,800	4,260,057	1085	2,441,949	1,162,095
**Chemotherapy**									
Both	98,246	185,853,696	88,445,604	17,125	32,397,412	15,417,550	4649	8,794,968	4,185,423
Men	48,101	91,059,408	43,334,109	8015	15,174,362	7,221,302	2170	4,107,295	1,954,614
Women	50,146	94,794,296	45,111,499	9110	17,223,050	8,196,248	2479	4,687,673	2,230,809
**Radiotherapy**									
Both	680	579,535	275,794	119	101,072	48,099	32	27,439	13,058
Men	321	276,443	131,556	54	46,122	21,949	15	12,565	5979
Women	359	303,092	144,238	65	54,951	26,150	18	14,875	7079
**Total**	141,987	281,926,655	134,165,603	24,750	49,147,216	23,388,586	6726	13,344,488	6,350,486

*PPP* Purchasing power parity in 2015–2017 (conversion factor 2.10); Colon cancer was defined as ICD C18; *TMRE* theoretical minimum risk exposure level scenario (population reaching ≥8000 MET-min/week); PA guidelines: population reaching at least 600 MET-min/week. ^a^Average of costs in 2015–2017

Direct costs with postmenopausal breast cancer (Int$ 226 million) represented 66% of all breast cancer costs (Table 3). We estimated that Int$ 26.9 million of postmenopausal breast cancer costs were attributable to lack of physical activity, which represents 12% of the postmenopausal breast cancer costs and 5.6% of all breast cancer costs. Breast cancer attributable costs were distributed as follows: Int$ 18.5 million for chemotherapy, Int$ 4.7 million for hospitalization, and Int$ 3.7 million for radiotherapy. About Int$ 4 million could be potentially saved annually by reaching PA guidelines, of which Int$ 2.6 million on chemotherapy, Int$ 676 thousand on hospitalizations and Int$ 539 thousand on radiotherapy.

**Table 3:** Attributable direct public health care procedures and costs for postmenopausal breast cancer by increasing physical activity in Brazil, 2015–2017^a^

	–	–		TMRE (≥8000 MET-min/week)			PA guidelines (≥600 MET-min/week)		
Procedures, sex	Total			Attributable			Attributable		
	Number of Procedures	Costs (R$)	PPP	Number of Procedures	Costs (R$)	PPP	Number of Procedures	Costs (R$)	PPP
**Hospitalization**									
Both	39,684	82,465,568	39,244,401	4717	9,793,987	4,660,844	687	1,420,173	675,844
Men	NA	NA	NA	NA	NA	NA	NA	NA	NA
Women	39,684	82,465,568	39,244,401	4717	9,793,987	4,660,844	687	1,420,173	675,844
**Chemotherapy**									
Both	1,116,395	327,324,128	155,769,731	132,562	38,885,592	18,505,199	19,230	5,647,669	2,687,660
Men	NA	NA	NA	NA	NA	NA	NA	NA	NA
Women	1,116,395	327,324,128	155,769,731	132,562	38,885,592	18,505,199	19,230	5,647,669	2,687,660
**Radiotherapy**									
Both	43,979	65,757,072	31,293,023	5223	7,808,938	3,716,182	758	1,133,530	539,433
Men	NA	NA	NA	NA	NA	NA	NA	NA	NA
Women	43,979	65,757,072	31,293,023	5223	7,808,938	3,716,182	758	1,133,530	539,433
**Total**	1,200,058	475,546,768	226,307,155	142,501	56,488,517	26,882,225	20,675	8,201,371	3,902,937

*PPP* Purchasing power parity in 2015–2017 (conversion factor 2.10); Post-menopausal breast cancer was defined as procedures and costs related to ICD C50 in women aged ≥50 years. *TMRE* theoretical minimum risk exposure level scenario (population reaching ≥8000 MET-min/week); *PA guidelines* population reaching at least 600 MET-min/week. ^a^Average of costs in 2015–2017

Combined costs of breast and colon cancer attributable to lack of physical activity was Int$ 50.3 million, of which Int$ 33.9 million were due to chemotherapy, Int$ 12.6 million to hospitalizations, and Int$ 3.8 million to radiotherapy. PA guidelines scenario would result in Int$ 10.3 million saved annually, of which Int$ 6.9 million were due to chemotherapy, Int$ 2.8 million to hospitalizations, and Int$ 552 thousand to radiotherapy.

## Discussion

In this study, we estimated the direct costs of colorectal and breast cancers in the Brazilian SUS attributable to lack of physical activity. We found that Int$ 26.9 million of postmenopausal breast cancer and Int$ 23.4 million of colon cancer costs were attributable to lack of physical activity in Brazil in 2017. Considering a plausible counterfactual scenario of reaching at least the physical activity guidelines would result in Int$ 10.3 million saved annually.

Cancer has an enormous societal cost. The increasing number of cancer patients has an escalating economic impact to public health systems and society. In 2016, it has been estimated that the cost of cancer to the Brazilian public and private health system was around Int$ 60 billion, which represented around 1.7% of the country’s Gross Domestic Product per year. Direct costs with inpatients and outpatients represent around 20% of all costs [19]. Our study showed that Int$ 553 million (12%) out of the Int$ 4.5 billion spent with direct health care related to all cancer in the Brazilian SUS were due to colorectal cancer (Int$ 212 million) and breast cancer (Int$ 341 million). Part of these costs could be saved or reallocated with investments in primary prevention strategies.

Quantifying the burden of cancer, in terms of cases, deaths and costs, attributable to modifiable risk factors can help policymakers to understand the importance of prioritizing primary prevention strategies. In Brazil, it has been estimated that about 27% of all cancer cases and 34% of all cancer deaths could be averted by reducing the prevalence of lifestyle risk factors such as smoking, alcohol consumption, unhealthy diet, overweight and obesity and lack of physical activity [20]. Annually, about 10 thousand cancer cases (3878 colon and 6712 breast) and 3226 cancer deaths (1444 colon and 1782 breast) could be potentially avoided by promoting physical activity [20]. Our study adds information to these previous estimates by quantifying the economic burden of breast and colorectal cancer attributable to lack of physical activity.

The relationship between physical activity and cancer have received great attention and sharply increased in the past few years [5]. Traditionally, physical activity has been associated with reduced risk of colon and breast cancer in postmenopausal women, as illustrated in the estimates from the Global Burden of Disease study [21]. However, more recently, large pooled data studies including over 1 million participants have suggested that physical activity may additionally reduce the risk of other types of cancer such as bladder, breast, endometrial, esophageal, stomach, glioma, kidney, lung, ovarian, pancreas and prostate [22]. Although the 2018 WCRF report considers convincing/probable the evidence for inverse association of physical activity with breast, colon and endometrial only [6], the American College of Sports Medicine (ACSM) recently considered strong the evidence for inverse association between physical activity and seven types of cancer: bladder, breast, colon, endometrial, esophageal, pancreas and stomach [23]. Due to these divergences in the literature, we decided to estimate the cost of breast and colorectal cancer, which are the most well-established, with available estimates of dose-response relationship with total physical activity [7].

To our knowledge, there have a few country-wide studies on the economic burden of cancer due to lack of physical activity [11, 24–26]. In the United Kingdom (UK), insufficient physical activity was responsible for £1.06 billion to the National Health Service in 2002, with breast and colon/rectum cancers contributing to £240 and £383 million, respectively [24]. Of note, the UK study included rectal cancers (C20) in their estimates, although there is limited evidence supporting that physical activity reduces this type of cancer [6]. A recent study conducted in the Sweden suggested that insufficient physical activity was responsible for 0.91% (1.7 billion Swedish Krona) of total health care costs in 2016, of which 575 million Swedish Krona were spent with health care utilization, mortality and early retirement due to breast and colon cancer [25]. Finally, in the United States of America, $0.38 billion were spent on direct costs of breast and $2.0 billion on colon cancers in 1995 due to lack of physical activity [26]. Although all studies were conducted in high-income countries, comparing cancer cost estimates from these studies is challenging due to its different methods, currencies, health care systems and year of reference.

The most comprehensive study estimated that insufficient physical activity cost health care systems Int$ 53.8 billion worldwide in 2013, of which Int$ 2.7 billion were spent on breast cancer and Int$ 2.5 billion on colon cancer [11]. Estimated direct health care costs for breast and colon cancer varied widely across the globe, from Int$ 16.7 million in African countries (breast, Int$ 8.8 million; colon, Int$ 7.9 million) to Int$ 5.2 billion (breast, Int$ 2.7 billion; colon, Int$ 2.5 billion) in the Western Pacific. In Brazil, direct health care costs for breast and colon cancer were Int$ 38.3 million and Int$ 36.4 million, respectively [11]. Our study provided similar, but more conservative estimates for the direct health care costs for breast and colon cancers. These differences might due to differences in data sources used to calculate PIF/PAF estimates, as well as health care costs. Of note, our study adds information providing direct costs due to hospitalizations, chemotherapy, and radiotherapy. In addition, we provided results for two alternative counterfactual scenarios (TMRE and physical activity guidelines).

Our study has several limitations. First, we considered only direct health care costs related to colorectal and breast cancers, which did not consider indirect costs (eg. premature mortality, loss of productivity and quality of life) and out-of-pocket expenditures. Physical activity may also reduce the risk of other types of cancer not included in our analysis [22]. Therefore, our findings should not be interpreted as the total costs of cancer attributable to lack of physical activity. Second, validated [27] but self-reported physical activity dates from 2013, when the most recent national representative health survey was conducted in Brazil. This may have introduced misclassification bias due to errors inherent to questionnaires and changes in physical activity over time. Third, we used RR estimates derived from a dose-response meta-analysis including studies mainly from US and Europe [7]. Transportability of RR, and therefore PIF/PAF estimates, may be biased if the prevalence of potential effect modifiers in these settings differs from Brazil [28]. Finally, SUS database gives only information on the total amount reimbursed by the federal government to the country’s health services, which did not consider other modalities of states’ and municipalities’ expenditures. Our cost estimates attributable to lack physical activity did not consider other non-communicable diseases previously linked with physical activity.

## Conclusions

Quantifying the economic burden of cancer in the public health system attributable to modifiable risk factors can help policymakers to understand and value the importance of primary prevention strategies. Our study provides evidence on the breast and colorectal cancers expenditures attributable to lack of physical activity in the Brazilian SUS. Annually, around Int$ 50.4 million of direct colorectal and breast cancers costs are attributable to lack of physical activity, which represents a substantial economic burden for the Brazilian health system. Primary prevention strategies aiming to promote physical activity, alongside with other health behaviors, are imperative to reduce the economic burden of cancer.

## Data Availability

All PNS data are available on the Brazilian Institute of Geography and Statistics (Instituto Brasileiro de Geografia e Estatística, IBGE) website at: http://www.ibge.gov.br/home/estatistica/populacao/pns/2013/default_microdados.shtm.

## Abbreviations

ACSM: American College of Sports Medicine
iInt$: International dollars
MET: Metabolic equivalent of tasks
Mi: Million
PAF: Population attributable fraction
PIF: Potential impact fractions
PPP: Purchasing power parity
R$: Reais
RR: Relative risks
PNS: National Health Survey *(Pesquisa Nacional de Saúde)*
SUS: Unified Health System (*Sistema Único de Saúde*)
TMRE: Theoretical minimum risk exposure level
WCRF: World Cancer Research Fund
WHO: World Health Organization
